# Linked family lives: the influences of siblings on Chinese adults’ wealth and health trajectories in later lives

**DOI:** 10.1093/geroni/igaf122.251

**Published:** 2025-12-31

**Authors:** Xinyi Chen, Wei-jun Jean Yeung

**Affiliations:** National Volunteer & Philanthropy Centre, Singapore, Singapore; Agency for Science, Technology and Research, Singapore, Singapore

## Abstract

Siblings are lifelong companions, providing both support and rivalry, yet their influence in later life remains underexplored in family research. This study examines how sibling number and gender shape the wealth and health trajectories of older adults in China, with a focus on gender differences in these relationships. Using four waves of China Health and Retirement Longitudinal Study (CHARLS) data and employing growth curve modeling, we find that having more siblings is generally associated with greater wealth and better physical and cognitive well-being in later life. While wealth and health decline with age, having siblings slows the trajectory of physical health decline but accelerates wealth reduction over time. Gender-specific analyses reveal that having sisters slows physical health decline, with stronger benefits for men than women. While males show greater resilience to age-related memory decline, having sisters weakens this advantage, whereas having brothers enhances it. These findings highlight the protective effects of sibling relationships on the physical and cognitive health of Chinese older adults. However, greater wealth decline among those with more siblings may stem from financial transfers for aging-related healthcare needs or diluted inheritance. Men also particularly benefit from sisters’ instrumental and emotional support, aligning with traditional caregiving expectations for women. As the one-child policy parent-generation enters later life in the coming decades, these findings suggest potential vulnerabilities in physical health trajectories and early-life wealth accumulation for their children, emphasizing the need for policy adaptations to support aging families.

